# Oxygen extraction ratio to identify patients at increased risk of intradialytic hypotension

**DOI:** 10.1038/s41598-021-84375-7

**Published:** 2021-02-26

**Authors:** Silverio Rotondi, Lida Tartaglione, Natalia De Martini, Domenico Bagordo, Sara Caissutti, Marzia Pasquali, Maria Luisa Muci, Sandro Mazzaferro

**Affiliations:** 1 Nephrology and Dialysis Unit, ICOT Hospital, Polo Pontino Sapienza University of Rome, Rome, Italy; 2 Department of Translational and Precision Medicine, Nephrology Unit at Policlinico Umberto I Hospital, “Sapienza” University of Rome, Viale del Policlinico 155, 00161 Rome, Italy; 3 Nephrology and Dialysis Unit, Policlinico Umberto I, Rome, Italy

**Keywords:** Nephrology, Renal replacement therapy, Haemodialysis

## Abstract

Intradialytic hypotension (IDH) is a hemodynamic phenomenon recently associated with decreased blood oxygen saturation (SO_2_). The ratio between peripheral oxygen saturation (SpO_2_) and central venous SO_2_ (ScvO_2_) or Oxygen Extraction Ratio (OER), which represents a roughly estimate of the amount of oxygen claimed by peripheral tissues, might be used to estimate haemodialysis (HD) related hypoxic stress. Aim of this pilot study was to evaluate the relationship between OER increments during dialysis sessions (ΔOER) and episodes of IDH. We enrolled chronic HD patients with permanent central venous catheter (CVC) and no fistula, in whom ScvO_2_ measurement is at hand. OER ([(SpO_2_ − ScvO_2_)/SpO_2_] × 100) was measured in three consecutive HD sessions (HD OER sessions) before HD, after 15′, 30′ and 60′ min and at the end of HD. Then, a one-year follow-up was planned to record the number of IDH episodes. In the 28 enrolled patients (age 74 ± 2.6 years), during 12 ± 1.2 months of follow up, incidence of IDH was 3.6%. We divided patients into two groups, above or below the median value of ΔOER at the end of HD, which was 36%. In these groups, the average incidence of IDH was 7% and 2% respectively (p < 0.01), while OER values before HD were not different. Notably, in the high ΔOER group the OER increment was evident since after 15′ and was significantly higher than in the low ∆OER group (∆OER-15′ = 19 ± 3.0% vs. 9.0 ± 3.0%; p < 0.05). By comparison, blood volume changes overlapped in the two groups (average change − 9 ± 0.8%). Values of ∆OER > 19% after only 15′ of HD treatment or > 36% at the end of the session characterize patients with higher rates of hypotension. Intradialytic ∆OER, a parameter of tissue hypoxic stress, identifies more fragile patients at greater risk of IDH.

## Introduction

Intradialytic hypotension (IDH) is a common hemodynamic phenomenon observed in haemodialysis (HD) patients, leading to a reduced tolerance of therapy. Patients typically report symptoms like abdominal discomfort, nausea, yawning, muscle cramps, restlessness and syncope. Since the definition of IDH is not uniform, its exact incidence, which varies from 7.5 to 69% according to the diagnostic criteria considered^1,2^, is not determined yet. However, most definitions include at least one of the following conditions: (1) reduction in blood pressure (BP) under specific thresholds, (2) intradialytic symptoms, (3) the need of medical intervention during haemodialysis session to restore blood volume (BV)^1^. In addition, IDH is associated with poor quality of life and higher mortality rate^3,4^. In clinical practice, intradialytic monitoring systems are able to assess patient hemodynamic parameters such as BP, BV and heart rate (HR). Nevertheless, these devices do not detect clinical conditions that might increase the risk for IDH and therefore, do not allow to adopt preventive strategies. Recently, a new non-invasive hemodynamic parameter measured by some dialysis machines: oxygen saturation, has been associated to episodes of IDH. This parameter must to have an important pathogenetic role in IDH but might also contribute to explain the increased mortality rate of HD patients. In fact, during HD sessions, both arterial oxygen saturation (SaO_2_) and central venous oxygen saturation (ScvO_2_, normal value 70–80%)^5^ decline in IDH prone patients, suggesting that either or both of these indices could be helpful predictors of IDH and fragility^6–8^. In particular, ScvO_2_ is lower in HD patients than in general population and its further reduction during HD could be secondary to the reduction of SaO_2_ (as in case of chronic lung diseases) but more likely to an increased parenchymal extraction of O_2_^7–9^. A more sensitive way to evaluate hypoxic phenomenon is by Oxygen Extraction Ratio (OER), i.e., the percentage of extracted oxygen in peripheral tissues, which is defined as the ratio between SaO_2_ and ScvO_2_. Major determinants of OER values are cardiac output, blood oxygen availability and peripheral oxygen consumption. Normal values are estimated to range between 20 and 30% in resting conditions, but can increase up to 70%, for example during cycling^10^. Thus, similarly to heart rate or breath frequency, OER values could provide an estimate of the individual cardiocirculatory and haemodynamic condition. However, due to the invasiveness of measuring ScvO_2_ few studies evaluate OER. In normal subjects, data have been reported during mountain ascending^11^ or while cycling^10^. In patients, OER has been used to evaluate the exercise capacity in adults with heart failure^12^ or the need of blood transfusions in anaemic children receiving cardiac surgery^13^. In intensive care Unit, OER is regarded as an index of parenchymal stress and oxygen consumption, and values > 50% are considered to be indicative of haemodynamic shock and worse prognosis^14,15^. Since OER measurement is handy in dialysis patients with central venous catheter (CVC), we recently evaluated it before and during HD sessions in a pilot study. We demonstrated that patients showing lower resting pre-HD OER values (< 32%) and/or greater OER increments (∆OER > 40%) after HD had higher mortality rate^16^. Also, in this same work, we could appreciate that OER values are rather stable and repeatable in the single patient, thus pointing to its potential usefulness to describe the hemodynamic capacity of a patient. As such, OER could theoretically represent a widely available, novel and simple tool to identify more fragile patients with significant subclinical HD-induced drop in cardiac output, tissue hypoxia and parenchymal stress, deserving increased attention. For all of these reasons, we considered useful to evaluate possible associations between OER change during HD (representative of cardiac output and development of subclinical ischemic injury) and IDH. To the best of our knowledge, no published paper correlates OER and IDH.

## Methods

We designed a prospective, single center (Nephrology Unit at Polo Pontino, Sapienza University of Rome), observational study (“*OER as a measurement of HD induced tissue hypoxia”*) involving HD patients receiving treatment thrice a week via permanent jugular central venous catheter (CVC). The study was approved in 2017 by the “Comitato Etico Lazio 2” EC (prot. N°107055/2017, https://www.aslroma2.it/COMITATOETICO/), was conducted in accordance with the declaration of Helsinki and received informed consent approval from all involved patients. Data for this study represent a sub-analysis aiming at evaluating the association between OER change during HD (ΔOER) and IDH.

Inclusion criteria were: patients age ≥ 18 years, undergoing chronic HD treatment since at least 3 months via permanent jugular CVC, with no evidence of acute underlying illness. Exclusion criteria were: less than 3 months of follow-up, presence of arteriovenous fistula^17^, evidence of displaced or malfunctioning CVC (checked with chest x-rays and CVC recirculation test), Chronic Obstructive Pulmonary Disease or peripheral oxygen saturation (SpO_2_) < 90% in resting condition. In addition, patients with severe refractory anemia (Hb < 9 g/dl despite adequate erythropoietin administration and iron supplement therapy), congestive heart failure (NYHA class ≥ II) and severe peripheral vascular ischemia were excluded.

The planned recruitment time was 3 months. In the first week after enrollment, we evaluated OER in three consecutive HD sessions (HD OER sessions) at 5 different time points: before HD, at 15, 30, 60 min since the beginning of HD and *post*-HD. For the purpose of statistical analysis, we considered the mean OER value obtained of the three HD OER sessions. During the follow-up, IDH episodes were recorded for each patient and for up to one-year. IDH was defined, according to K/DOQI guidelines, as the occurrence of either/or any of the following: (1) a decrease in systolic blood pressure > 20 mmHg, or a decrease in mean arterial pressure > 10 mmHg; (2) the presence of symptoms of end-organ ischemia; (3) the need for intervention carried out by the dialysis staff^18^.

OER and delta OER (ΔOER) were calculated using the following formula:

$$ {\text{OER }} = [({\text{SpO}}_{{2}} - {\text{ ScvO}}_{{2}} )/{\text{SpO}}{}_{{2}}] \, \times { 1}00. $$

$$ \Delta {\text{OER }} =  \left[ {\left( {{\text{OER}}_{{{\text{Tx}}}} - {\text{OER}}_{{{\text{T}}0}} } \right)/{\text{OER}}_{{{\text{T}}0}} } \right)] \, \times { 1}00. $$

where T0 is pre-HD OER and Tx is the value obtained at the established times.

SpO_2_ was monitored via peripheral pulse-oximetry device (Max Puls Two, Eurosanitas Italy; accuracy of SpO_2_ measurement = 2%). Patients wore the finger oximeter during HD OER sessions, and SpO_2_ values were recorded at the established times. Pre-HD ScvO_2_ was sampled from the arterial line of the CVC after discarding 20 ml of blood and before connection to the extracorporeal circuit, while, during dialysis, blood was sampled from the arterial line of the dialysis circuit. Blood gas analysis was immediately performed with a dedicated equipment (GEM 4000 premier, Instrumentation Laboratory Italy; accuracy of SO_2_ measurement = 2%). In order to avoid pre-analytical artifacts, handling of blood for gas analysis was standardized according to the manufacturer′s instructions.

All patients received standard bicarbonate dialysis, according to their individual prescriptions of electrolyte concentrations as well as blood and dialysate flows. The HD OER sessions lasted 4 h and during these sessions patients were fasting. Dialyzer membrane was polyaryletheresufone in all, with surface tailored to the patient body surface. Blood volume changes were measured by the optical probe with which the Artis dialysis machine (Baxter srl) is equipped and which is located on the arterial line of the extracorporeal circuit. Blood volume changes are derived from changes in hemoglobin concentration. All patients were connected to the extracorporeal circuit without initial hemorrhage. To increase tolerance to treatment, it is standard policy of our Center to keep ultrafiltration rate at ≤ 10 ml/kg/h and maintain a dialysate temperature between 35.5 and 36.0 °C.

### Statistical analysis

Since this is a pilot study for purely exploratory purposes, a formal calculation of the sample size was not carried out. We arbitrarily decided to enroll a minimum of 20 patients. Data are expressed as mean ± standard error (SE) for Gaussian variables. We used the Shapiro test to evaluate normality of continuous measurements. Chi-squared test was used for qualitative variables. Significant statistical differences among various predictors and time were evaluated by mixed-effects linear regression analysis and repeated measures ANOVA and multiple tests were adjusted through Bonferroni correction. The global level for statistical significance was pre-specified as 5%. T-test or parametric ANOVA were used to compare measurements among groups for quantitative variables. Bonferroni's adjusted pairwise comparisons were used to compare groups in pairs if parametric ANOVA was significant. All tests were two tailed and (adjusted) p values < 0.05 were considered as statistically significant.

Analyses were performed using the open-source software package R, 3.4.0 version.

### Ethical standards

All the procedures involving humans in this study were in agreement with common and standard clinical practice and in accordance with institutional and/or national research committee and with the 1964 Helsinki Declaration and its later amendments or comparable ethical standards.

## Results

**Figure 1:**
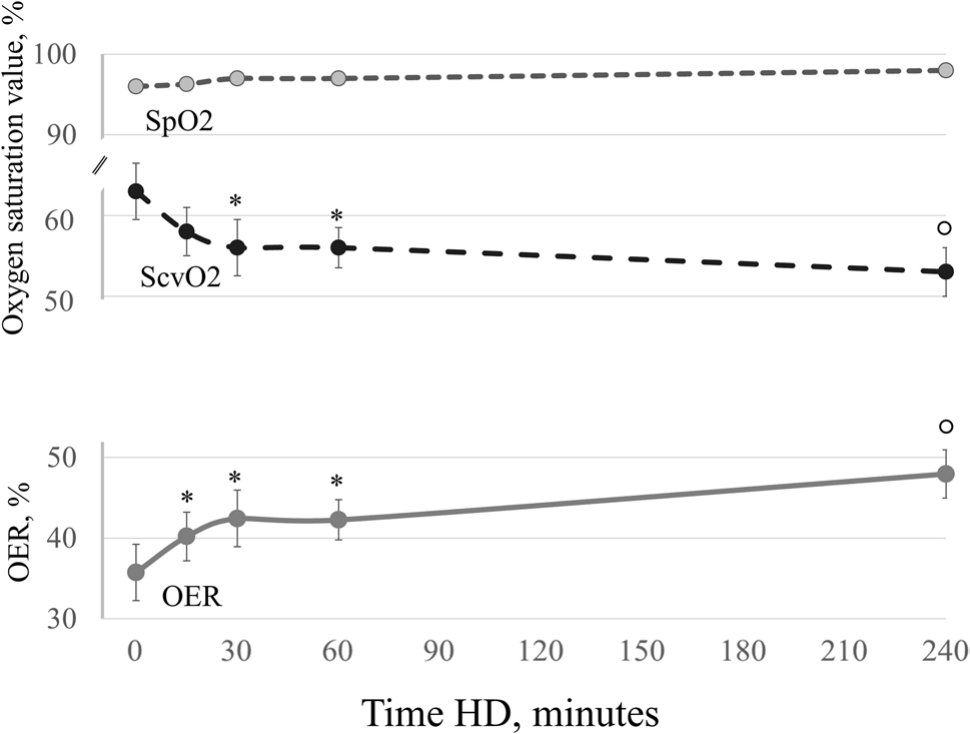
Oxygen saturation trends during dialysis. In the population as a whole, SpO_2_ was stable, while ScvO_2_ decreased after 30 min and OER increased after 15 min of HD initiation (repeated measures ANOVA: p < 0.001 for both variables). Bonferroni post-hoc test vs. basal values: *p < 0.01; °p < 0.001. SpO_2_: peripheral oxygen saturation; ScvO_2_: central venous oxygen saturation; OER: oxygen extraction rate; HD: hemodialysis.

Between 01/01/2017 and 31/03/2017, from a total of 32 patients receiving haemodialysis with a permanent jugular CVC, 28 (13 males and 15 females, aged 74 ± 2.6 years, receiving replacement therapy since an average of 46 ± 6.5 months) fulfilled the inclusion criteria and consented to be enrolled for the study. Four patients (65 ± 9 years, 2 males and 2 females) were excluded, for the presence of arteriovenous fistula (two), for SpO_2_ < 90% in resting condition (one) and for less than three months of follow-up (one). Thus 28 patients, whose clinical and biochemical characteristics are shown in Table 1, were followed up for 12 ± 1.2 months to record IDH episodes (study closed on 31/03/2018). As reported in the table, two patients were obese (10%), eleven had diabetes mellitus (39%) and twenty-six had vascular co-morbidities (93%). In addition, 93% had hypertension while history of ischemic heart disease and peripheral vasculopathy were present in 50% and 61% of the patients, respectively. During the three HD sessions for OER behavior determination, all patients remained asymptomatic. Pre HD OER values and the intradialytic OER trends overlapped in the three HD OER sessions (supplementary material Figure 1). The maximum calculated difference of pre HD OER in the individual patient was 5.5%, in agreement with previously published data showing a maximum individual variability of 6.6%^16^. In the whole population pre-HD OER averaged 34 ± 1.4%, and reached 46 ± 1.8% post-HD, with a resulting average ∆OER of 39 ± 5% (Table 1). SpO_2_ did not change during HD OER sessions (Fig. 1), while ScvO_2_ decreased significantly from 63 ± 3.5 to 53 ± 3.0% (Fig. 1 p < 0.001). In the three HD OER sessions, the average reduction of BV was − 9 ± 0.8%. The intradialytic increment of OER was significant since after 15 min (OER 15′: 40 ± 1.2%; p < 0.001 vs pre-HD OER) and was followed by a progressive increase up to the final post-HD value (p < 0.0001) (Fig. 1). During follow up, we monitored 4342 HD sessions with 188 episodes of IDH (defined as above), which were recorded at least once in 24/28 patients, and produced a median incidence of 3.6% episodes (range between 0 and 15%).

**Table 1:** Clinical and biochemical characteristics of enrolled patients.

Clinical and biochemical characteristics (n 28)	
Male/female, n (%)	13 (46)/15 (54)
Age, years	74.4 ± 2.6
HD vintage, months	46.5 ± 6.5
BMI	26.4 ± 1.2
Diabetes mellitus, n (%)	11 (39%)
Hypertension, n (%)	26 (93%)
Vascular co-morbidities^a^, n (%)	26 (93)
Systolic BP, mmHg	127 ± 3.6
Diastolic BP, mmHg	69 ± 2.2
MAP, mmHg	88 ± 2.4
HR, bpm	75 ± 5.2
Hb, g/dl	10.2 ± 0,4
CRP, mg/dl	2.2 ± 0.5
ESR, mm	36 ± 4.7
Ferritin, mcg/l	174 ± 12
Albumin, g/dl	3.1 ± 0.1
Na, meq/l	141 ± 3.1
K, meq/l	5.5 ± 1.1
HCO_3_^−^, meq/l	23.4 ± 4.2
Ca, mg/dl	8.4 ± 0.1
P, mg/dl	5.3 ± 0.2
PTH, pg/ml	311 ± 30
UF, ml/h/kg	7 ± 0.6
ScvO_2_ pre-HD, %	63 ± 3.5
ScvO_2_ post-HD, %	53 ± 3.0
SpO_2_ pre HD, %	96 ± 0.5
SpO_2_ post HD, %	98 ± 0.4
OER pre-HD	34 ± 1.4
OER post-HD	46 ± 1.8
∆OER, %	39 ± 5.0
BV post-HD, %	− 9 ± 0.8

Data are expressed as mean ± SE.

HD: haemodialysis; BMI: body mass index; BP: blood pressure; MAP: mean arterial pression; HR: heart rate; Hb: haemoglobin; CRP: C-reactive protein; ESR: erythrocyte sedimentation rate; Na: sodiemia; HCO_3_^−^: bicarbonate; Ca: calcemia; P: phosphatemia; PTH: parathormone; UF: ultrafiltration rate; ScvO_2_: central venous SO_2_; SpO_2_: peripheral oxygen saturation; OER: oxygen extraction ratio; **∆**OER: variation in OER; BV: blood volume.

^a^Vascular co-morbidities: hypertension, ischemic heart disease, peripheral vasculopathy.

**Figure 2:**
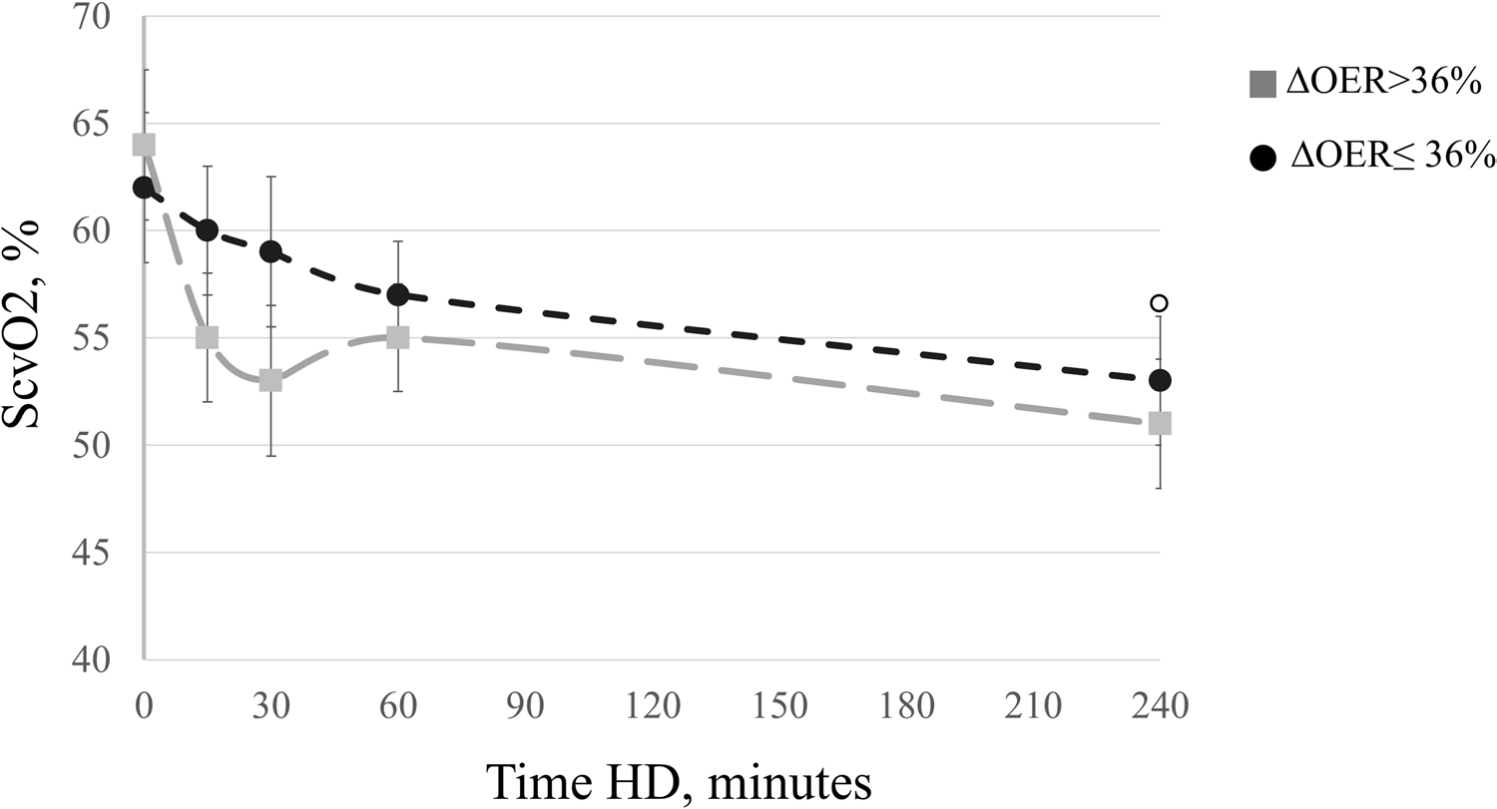
Comparison of ScvO_2_ trends during HD between the two groups ΔOER > 36% and ΔOER ≤ 36%. ScvO_2_ decreased significantly (ANOVA, p < 0.001) in both groups but without differences between them, in particular after 30 min. ScvO_2_: central venous oxygen saturation; ∆OER: percent change in OER; HD: haemodialysis.

**Figure 3:**
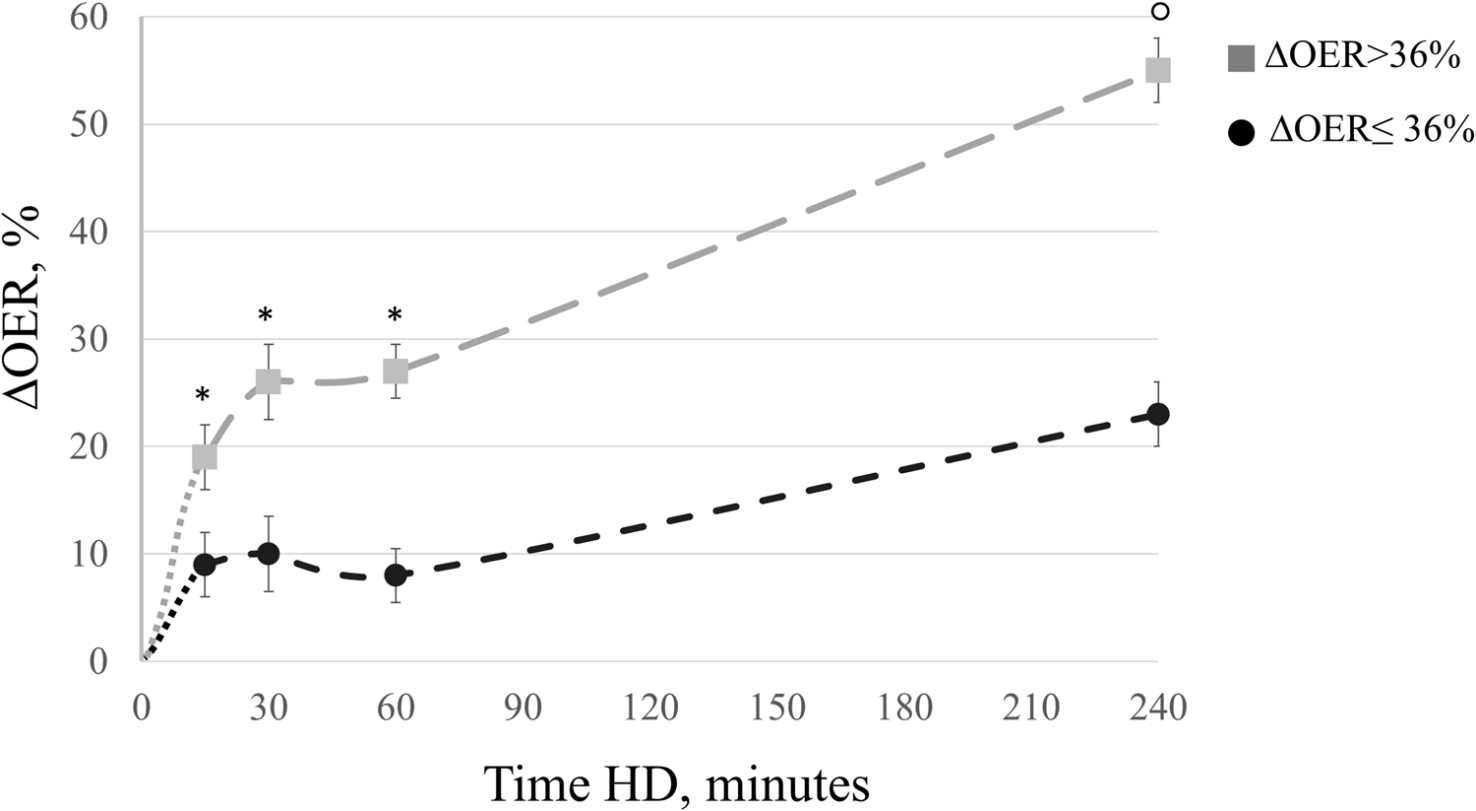
During HD ∆OER increased in both groups with either low or high ΔOER (repeated measures ANOVA, p < 0.001), but in the ΔOER > 36% group the increment was significantly higher than in the ΔOER ≤ 36% group after 15 min, 30 min and 60 min. Bonferroni post-hoc test, ΔOER > 36% vs. ΔOER ≤ 36% *p < 0.05; °p < 0.001. ∆OER: percent change in OER; HD: haemodialysis;

**Figure 4:**
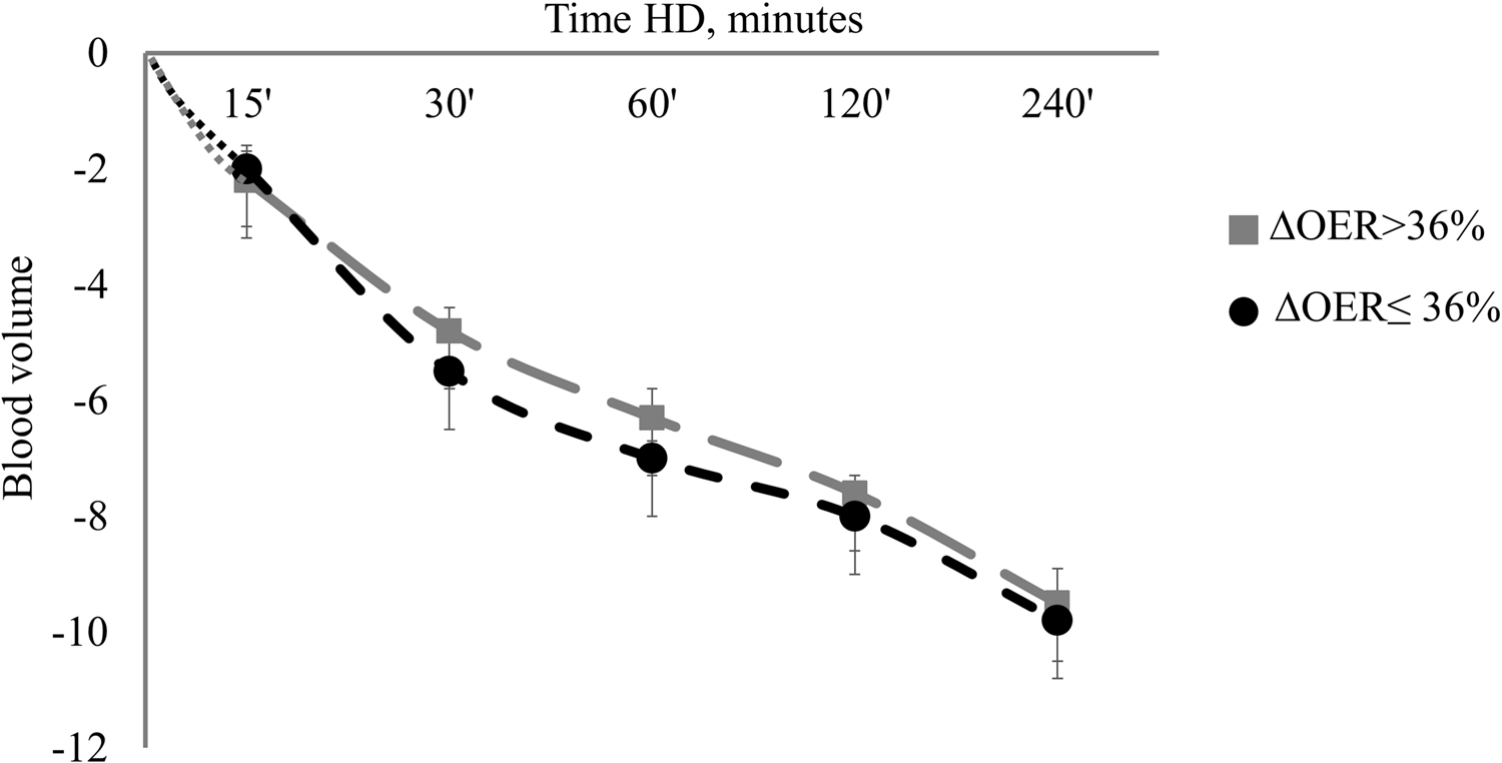
Overlapping trends of hemodialysis induced changes in blood volume in ΔOER > 36% and ΔOER ≤ 36% groups.

To assess the link between ∆OER and IDH, we divided patients into two groups according to the median ∆OER value (threshold at 36%). As shown in Table 2, these two groups had similar age (76 ± 2.4 vs 73 ± 3.0 years; p = n.s.), HD vintage (52 ± 8.6 vs 40 ± 4.0 months; p = n.s.), systolic BP (125 ± 3.2 vs 129 ± 4.0 mmHg; p = n.s.), diastolic BP (67 ± 2.2 vs 70 ± 2.2 mmHg; p = n.s.), HR (70 ± 2.2 vs. 76 ± 2.3 bpm; p = n.s.) and other biomarkers of anaemia, inflammation and pre- and post-HD values of electrolytes. Before and after HD, ScvO_2_ values were non-significantly different between the groups (respectively, 62 ± 3.5 vs 66 ± 3.0; p = n.s. and 53 ± 3.0 vs 51 ± 3.0; p = n.s.), similarly to the intradialytic trend (Table 2 and Fig. 2). As for OER, pre-HD values were not different between the groups (Table 2), but during session the percent increment was significantly higher since after 15 min in the high *∆OER* compared to the low *∆OER* group (19 ± 3.0% vs. 9.0 ± 3.0% respectively; p = 0.009), a difference that persisted after 30 min (26 ± 3.0% vs. 10 ± 4.0%; p = 0.037), and 60 min of treatment (27 ± 4.0% vs. 8 ± 4.6%; p = 0.001) (Fig. 3). By comparison, blood volume reduction overlapped in these two groups (− 9 ± 0.4 vs − 9 ± 1.6 p = n.s.) (Fig. 4).

**Table 2:** Characteristics of patients divided according to the median ΔOER% final value.

Characteristics of patients divided according to the median ΔOER% final value (threshold 36%)			p
	ΔOER ≤ 36% (n 14)	ΔOER > 36% (n 14)	
Age, years	76.4 ± 2.4	73.3 ± 3.0	0.440
HD vintage, months	52.2 ± 8.6	40.3 ± 4.0	0.218
Diabetes mellitus, n (%)	5 (36)	6 (42)	0.815
Hypertension, n (%)	12 (85)	14 (100)	0.820
Vascular co-morbidities^a^, n (%)	13 (93)	13 (93)	0.999
Anti-hypertensive drugs, n(%)	12 (85)	14 (100)	0.820
Pre-HD systolic BP, mmHg	125 ± 3.2	129 ± 4.0	0.401
Pre-HD diastolic BP, mmHg	67 ± 2.2	70 ± 2.2	0.341
Post-HD systolic BP, mmHg	131 ± 3.0	130 ± 3.0	0.816
Post-HD diastolic BP, mmHg	71 ± 2.2	73 ± 2.2	0.526
Pre-HD HR, bpm	74 ± 4.8	76 ± 5.6	0.870
Post-HD HR, bpm	72 ± 5.0	71 ± 4.5	0.921
UF, ml/h/kg	7 ± 0.6	6 ± 0.6	0.062
UF total, L	2.0 ± 0.1	2.2 ± 0.5	0.820
Hb, g/dl	10.1 ± 0,4	10.3 ± 0,2	0.850
CRP, mg/dl	2.2 ± 0.5	2.1 ± 0.6	0.820
ESR, mm	36 ± 4.7	34 ± 5.0	0.630
Albumin, g/dl	3.1 ± 0.1	3.0 ± 0.2	0.880
Na pre-HD, mmol/l	140 ± 2.2	141 ± 3.5	0.921
Na post-HD, mmol/l	139 ± 1.8	140 ± 3.0	0.890
K pre-HD, mmol/l	5.6 ± 1.6	5.4 ± 1.0	0.860
K post-HD, mmol/l	3.5 ± 0.8	3.4 ± 0.7	0.880
HCO_3_^–^ pre-HD , mmol/l	22.3 ± 3.8	23.4 ± 4.5	0.845
HCO_3_^–^ post-HD , mmol/l	27.2 ± 1.3	26.5 ± 1.4	0.900
HD treatments, n	2197	2145	
IDH episodes, n	64	124	
IDH episodes, %	2	7	00.01
Follow-up time, months	12 ± 1.0	12 ± 1.1	0.060
ScvO_2_ pre-HD, %	62 ± 3.5	66 ± 3.0	0.780
ScvO_2_ post-HD, %	53 ± 3.0	51 ± 3.0	0.720
SpO_2_ pre HD, %	97 ± 0.5	96 ± 0.5	0.830
SpO_2_ post HD, %	98 ± 0.4	98 ± 0.4	0.920
OER pre-HD	36 ± 1.6	30 ± 1.5	0.055
OER post-HD	45 ± 1.7	47 ± 1.6	0.399
∆OER, %	23 ± 3.0	55 ± 3.6	0.001
BV post HD, %	− 9 ± 1.6	− 9 ± 0.4	0.990

Data are expressed as mean ± SE.

Chi-squared test for qualitative variables and T-test for quantitative variables were used to compare measurements between groups.

∆OER: variation in OER; IDH: intradialytic hypotension; HD: hemodialysis; BP: blood pressure; HR: heart rate: UF: ultrafiltration rate; Hb: hemoglobin; CRP: C-reactive protein; ESR: erythrocyte sedimentation rate; ScvO_2_: central venous SO_2_ SpO_2_: peripheral oxygen saturation; OER: oxygen extraction ratio; BV: blood volume.

^a^Vascular co-morbidities: hypertension, ischemic heart disease, peripheral vasculopathy.

**Figure 5:**
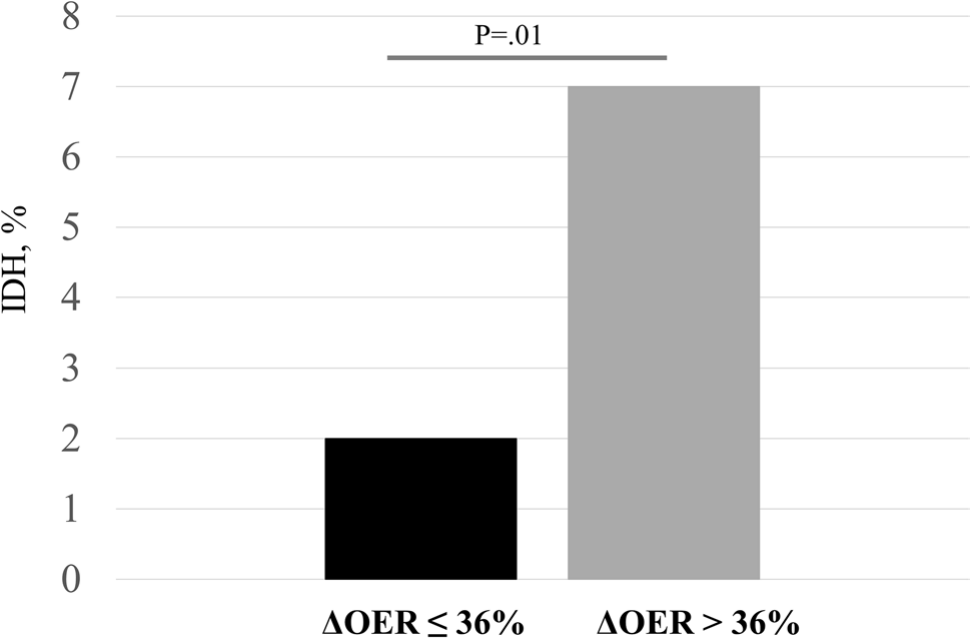
The ΔOER > 36% group had higher IDH incidence than ΔOER ≤ 36% group. Bonferroni test p  0.01. ∆OER: percent change in OER; IDH: intradialytic hypotension.

During follow-up, which lasted 12 ± 1.0 months in both groups and included a total of 2197 and 2145 HD sessions in the low and high ∆OER groups respectively, IDH events were 64 (2.0%) in the former and 124 (7%) in the last (p < 0.01) (Table 2; Fig. 5). Moreover, if we divided patients according to the median IDH incidence (threshold 3.6%) the higher incidence group had greater post-HD ∆OER % than the lower incidence group (44 ± 3.6 vs. 35 ± 3.0%; p 0.044, respectively, supplementary material Table 1).

By comparison, if we divided patients according to the median BV reduction, no difference emerged in the prevalence of IDH (supplementary materials Table 2).

## Discussion

Our data show that intradialytic ∆OER, a measurement of tissue hypoxic stress differs in patients with different rates of IDH. In particular, a percentage increment > 19% since after 15 min of treatment or a final increment > 36% could be helpful to identify patients at higher risk of hemodynamic instability. The pathogenesis of IDH is multifactorial and involves mechanism responsible for a drop in cardiac output, like ultrafiltration rate, electrolyte shifts, and biocompatibility of solutions and/or of membranes. Other recently discovered biomarkers of mineral and bone disorders now regarded as additional long-term cardiovascular risk factor in HD patients^19,20^ could be at play, but their acute effects are not still evaluated. At variance, during each HD session the rapid hemodynamic changes produced by dialysis inevitably induce an acute hypoxic stress that in some patients becomes clinically relevant thus representing a further burden to the cardiovascular system. These episodes are secondary to reduced cardiac output with the eventual low oxygen delivery and increased parenchymal tissues demand^6,17^. Both ultrafiltration rate and the dialytic procedure per se can lead to parenchymal stress responsible for IDH^16,21–25^. In our study, in the three HD OER sessions monitored at enrollment, OER increased progressively in all patients with an average end of dialysis ∆OER of 39% (Fig. 1). This is in perfect agreement with the available evidence^16^ of HD related hypoxic stress which is not sensed by the patients and is not detected by commonly available parameters like BP and HR^6,7,16,26–28^. Therefore, ∆OER seems to offer a prompt and direct measurement of the hypoxic stress produced by HD and could thus represent a useful tool to identify more fragile patients. As predictable, also ScvO_2_ changed during HD (significant drop after 30 min, Fig. 1) but later than OER which increased since after 15 min, thus resulting more sensitive. Notably, in our study the incidence of IDH (3.6%) was significantly lower than the average 20% most frequently reported in the literature^29,30^. This low incidence of IDH in our population can be explained by our standard policy of keeping ultrafiltration rates at ≤ 10 ml/kg/h and dialysate temperature at 35.5–36.0 °C to increase hemodynamic stability^31,32^. Therefore, we can underline that ∆OER was sensitive even in a population with low prevalence of IDH. Also, in our study, the two groups of patients obtained according to the median ΔOER threshold had similar prevalence of comorbidities and similar HD related parameters (like UF rates, BP, HR etc. see Table 2). Notably, pre-HD OER values, which were not different between the two groups (36 ± 1.6 vs 30 ± 1.5 respectively in the low- and high- ∆OER groups), were on average higher than the normal reference of 20% in resting conditions, suggesting that uremic patients have an increased oxygen requirement even when inactive. In addition, in the high *ΔOER* group with greater incidence of IDH (Fig. 5), OER increment was remarkably greater than the more stable group after only 15 min (19% vs 8%, respectively) (Fig. 3). Therefore, since we measured OER during three consecutive and asymptomatic HD sessions, values of ∆OER > 19% since after 15 min could be an early and sensitive indicator of hypoxic stress, capable of identifying cases at increased risk of IDH. Our results also describe how the response to the hypoxic stress can be different among patients. In particular, the increment of OER was rather smooth and progressive in the *ΔOER* ≤ *36%* group and sharp and rapid in the *ΔOER* > 36% group, suggesting a maladaptive response in the latter. Thus, also OER trends could be helpful to recognize patients that are more sensitive to the hemodialysis-related cardio-circulatory stress.

A major limitation of OER measurement technique is that it can be exclusively applied to patients with CVC and without a fistula. However, CVC use in HD, although not recommended, is nonetheless necessary in many patients with poor vascular system, who are also fragile and with a higher risk of IDH and mortality rate. Therefore, in this population, the implementation of a new hemodynamic parameter measurable during HD session, such as OER, could be useful in clinical practice. Another limitation of our study is the small number of patients enrolled. However, this was a pilot study and it is important that differences have been detected even with such a low number of cases and in a population with a low prevalence of IDH episodes, using a cheap, non-sophisticated and commonly available equipment. A multicenter study including hundreds of patients is now warranted to confirm our results and to evaluate the association with other outcomes like quality of life and overall and cardiovascular mortality**.** Also, in a next future, we need to know how much resting values of OER are stable over time in the individual patient. Our data from a previous study^16^ suggest a significant stability in the short term; however, we need to characterize how much resting and HD induced changes in OER are affected by occasional or chronic clinical events.

In conclusion, measurement of ∆OER as early as 15 min after initiation or at the end of HD sessions could be a new and simple way to identify hemodynamically fragile patients. Efforts are warranted to test if adopting preventive measures in patients identified with OER changes, effectively allow to reduce the rate of IDH.

## Supplementary Information

Supplementary Information.
