# Supplementary material for: Oxygen extraction ratio to identify patients at increased risk of intradialytic hypotension
Source: Sci Rep. 2021 Feb 26;11:4801. doi: 10.1038/s41598-021-84375-7 (PMC7910620; doi:10.1038/s41598-021-84375-7)
Supplement: Supplementary file 1 — Supplementary Information. [file 41598_2021_84375_MOESM1_ESM.pdf]

Oxygen Extraction Ratio to identify patients at increased risk of intradialytic hypotension

Silverio Rotondi[1], Lida Tartaglione[1], Natalia De Martini[2], Domenico Bagordo[2], Sara Caissutti[1], Marzia Pasquali[3], Maria Luisa Muci[1] AND Sandro Mazzaferro[2]

[1] Nephrology and Dialysis Unit, ICOT Hospital, Polo Pontino Sapienza University of Rome.

[2] Department of Translational and Precision Medicine, Nephrology Unit at Policlinico Umberto I Hospital, Sapienza University of Rome, Viale del Policlinico 155, 00161 Rome, Italy

[3] Nephrology and Dialysis Unit, Policlinico Umberto I, Rome. Italy

Corresponding Author: Prof. Sandro Mazzaferro, “Sapienza” University of Rome

Address: Viale del Policlinico 155 00161, Rome

Tel: 0039 06 49978393

Fax: 0039 06 49978393

E-mail: sandro.mazzaferro@uniroma1.it

Key words: Intradialytic hypotension, Oxygen Extraction Ratio, Arterial oxygen saturation, central venous oxygen saturation

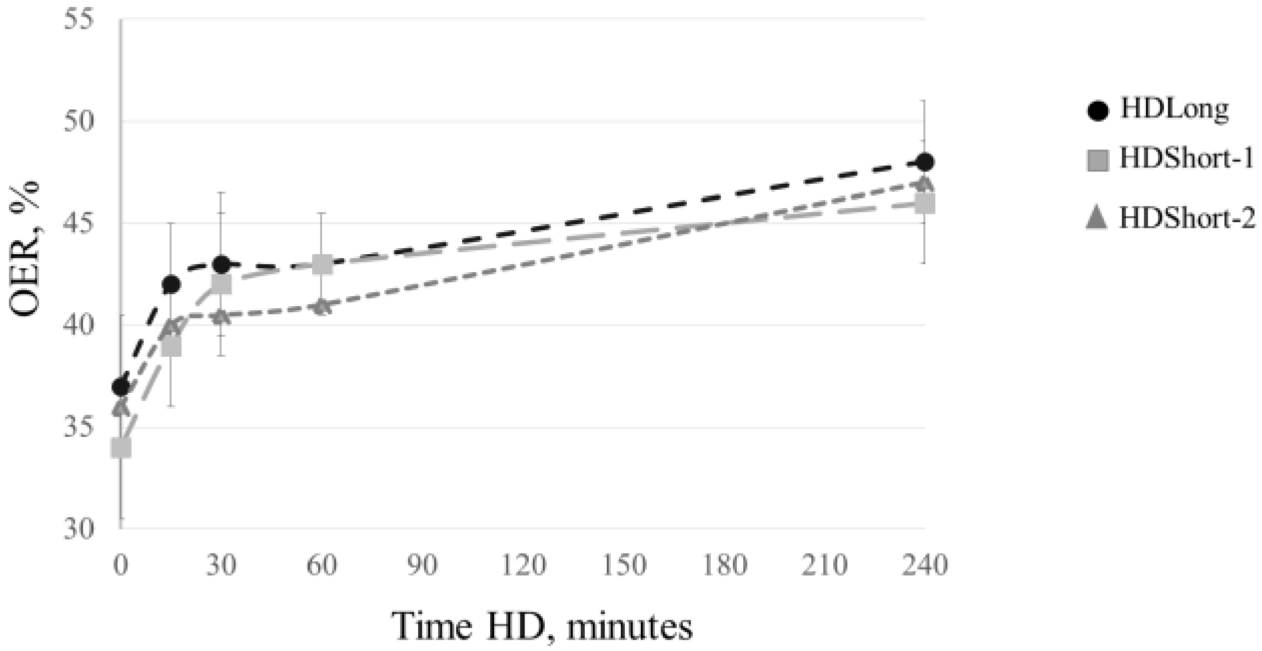

Supplementary material Figure 1: OER values consistently overlapped before and during treatment in the Long- and Short- interval HD sessions (indicated as 1 and 2).
HD Long = Hemodialysis following long interdialytic interval; HDShort = Hemodialysis following short interdialytic interval; OER: oxygen extraction ratio

Patients characteristics in the two groups identified according to the median IDH incidence % (threshold 3.6%)			p
	IDH ≤ 3.6%, (n.14)	IDH > 3.6%, (n.14)	
Age, years	76 ± 2.4	73 ± 3.0	.440
HD vintage, months	52 ± 8.6	40 ± 4.0	.218
Pre-HD Systolic BP, mmHg	125 ± 3.2	129 ± 4.0	.401
HD treatments, n	2431	1911	
IDH episodes, n	27	159	
IDH episodes, %	0.9	9	.001
Follow-up time, months	13 ± 1.0	10 ± 1.1	.060
OER pre-HD	34 ± 1.6	33 ± 1.5	.652
OER post-HD	45 ± 1.7	47 ± 1.6	.399
∆OER, %	35 ± 3.0	44 ± 3.6	.044
UF, ml/h/kg	8 ± 0.6	6 ± 0.6	.062
UF total, L	2.1 ± 0.1	2.2 ± 0.5	.820

Supplementary material Table 1. Characteristics of patients divided according to the median IDH incidence.

Data are expressed as mean ± SE. IDH: intradialytic hypotension; HD: hemodialysis; OER: oxygen extraction ratio; ∆OER: variation in OER. Chi-squared test for qualitative variables and T-test for quantitative variables were used to compare measurements between groups

Incidence of IDH and OER values in patients divided according to the median Blood Volume (BV) reduction (threshold -9,4 %)			
	B.V > -9,4% (n.14)	B.V ≤ -9,4%(n.14)	p.
Age, years	75.4 ± 2.4	74.3 ± 3.0	.777
HD vintage, months	48.2 ± 7.0	44.2 ± 6.0	.652
BV post HD, %	-6 ± 1.6	-11 ± 0.4	.001
HD treatments, n	2100	2242	
IDH episodes, n	84	76	
IDH episodes, %	4.0	3.4	.700
Follow-up time, months	14 ± 1.0	13 ± 1.1	.741
OER pre-HD	33± 2.0	32 ± 1.8	.713
OER post-HD	46 ± 2.0	45 ± 1.6	.699
ΔOER, %	37 ± 3.0	37 ± 3.6	1.00
UF, ml/h/kg	6 ± 0.6	7 ± 0.6	.249
UF total, L	2.0 ± 0.1	2.2 ± 0.5	.698

Supplementary material Table 2. Characteristics of patients divided according to the median B.V. % final value.

Data are expressed as mean ± SE. ΔOER: variation in OER; IDH: intradialytic hypotension; HD: hemodialysis; UF: ultrafiltration rate; OER: oxygen extraction ratio. Chi-squared test for qualitative variables and T-test for quantitative variables were used to compare measurements between groups.
